# Budget impact of intravenous iron therapy with ferric carboxymaltose in patients with chronic heart failure and iron deficiency in Germany

**DOI:** 10.1002/ehf2.12179

**Published:** 2017-07-03

**Authors:** Ulrike Theidel, Saku Väätäinen, Janne Martikainen, Erkki Soini, Thomas Hardt, Wolfram Doehner

**Affiliations:** ^1^ Xcenda GmbH Hannover 30159 Germany; ^2^ ESiOR Oy Kuopio 70100 Finland; ^3^ Vifor Pharma Deutschland GmbH Munich 81379 Germany; ^4^ Center for Stroke Research and Department of Cardiology Charite Universitätsmedizin Berlin Berlin 13353 Germany

**Keywords:** Iron deficiency, Chronic heart failure, Cost, Budget impact, Ferric carboxymaltose, Ferinject

## Abstract

**Aims:**

Treatment of iron deficiency (ID) in patients with heart failure (HF) with intravenous iron substitution [ferric carboxymaltose (FCM)] has previously shown significant improvements in exercise capacity, New York Heart Association (NYHA) functional class, quality of life, and reduction of hospitalization. The aim of this study was to estimate the budget impact of FCM treatment for patients with HF and ID.

**Methods and results:**

Individual patient data from four double‐blind randomized controlled trials were pooled for this analysis. Expected outcomes were modelled for a treatment period of 1 year, using multivariate statistical methods. Associated unit costs were derived from claims data. Budget impact was calculated from the perspective of the Statutory Health Insurance. Multiple deterministic sensitivity analyses were performed. The annual budget impact for therapy with FCM vs. no‐iron therapy was €2 735 505 and €2 695 474 for 1000 patients, respectively, resulting in additional annual costs of €40.03 for each treated patient. Main costs drivers are the FCM treatment cost and cost of hospitalizations due to HF worsening. FCM therapy compared with no‐iron therapy resulted in reduced cost per 1000 patients: for reduced hospitalization due to HF worsening (52 vs. 129 hospitalizations amounting to €230 591 vs. €597 078), for reduced other medication (€1 611 007 vs. €1 679 908), fewer outpatient visits (€332 523 vs. €378 019), and home visits (€29 627 vs. €40 469). Sensitivity analyses showed robustness of the results.

**Conclusions:**

Therapy with FCM has a minimal budget impact of €40 031 per 1000 patients per year. This budget impact translates into reduced and shorter hospitalizations and improved symptomatic status of the patients.

## Introduction

Chronic heart failure (CHF) is a debilitating and life‐threatening chronic disease, where the heart fails to deliver oxygen to fulfil metabolism tissues requirements. Symptoms (breathlessness, fatigue, and fluid retention) appear slowly and worsen over time,^1^, ^2^ which leads to a significant impact on patient's quality of life.^3^, ^4^, ^5^

In Germany, it is estimated that 1.6 million patients suffer from CHF with 200 000–300 000 incident cases per year and increases with age.^6^ CHF is the most common reason for hospital admissions with increasing numbers over the past 10 years from 306 736 in 2005 to 432 893 in 2014.^7^ In 2014, CHF was in absolute numbers, the fourth most frequent cause of death, affecting 5% of all deaths in Germany.^8^

Economically, CHF is responsible for 1% to 2% of direct healthcare costs in the Western industrialized nations and for around 1.1% in Germany.^9^ Studies of the German Competence Network Heart Failure showed that patients with CHF have 6.14 (±9) annual contacts to their general practitioner, but 72% of all medical costs are for hospital admissions.^10^ Overall, treatment of CHF causes 2.3 times higher cost compared with the average insured individuals of the Statutory Health Insurance (SHI) in Germany.^11^

Approximately 50% of patients with CHF are iron deficient.^12^, ^13^, ^14^ Iron deficiency (ID) is defined as a ferritin level <100 μg/L or ferritin 100–299 μg/L with a transferrin saturation <20%.^12^ It is a progressive condition, which can be linked to worsening symptomatic conditions and is a poor prognostic sign, independently of other prognostic factors. In CHF, ID is related to the severity of disease and may be present with or without the clinical occurrence of anaemia. Recent ESC guidelines for the diagnosis and treatment of heart failure (HF) confirm ID being an important comorbidity and recommend that treatment specifically with ferric carboxymaltose (FCM) should be considered in symptomatic patients with systolic CHF and ID.^15^

Randomized placebo‐controlled trials (RCT) with FCM have shown a clinical benefit of the correction of ID in patients with CHF.^16^, ^17^, ^18^, ^19^ Compared with placebo (no‐iron), treatment with FCM is associated with significant improvements in functional capacity, symptoms, and quality of life. These improvements are related to a reduction in the risk of first hospitalization as well as recurrent hospitalization due to worsening HF in patients with systolic CHF+ID.

The aim of this study is therefore to evaluate the net budget impact of treatment with FCM in patients with CHF and ID in the German public healthcare setting compared with no‐iron treatment.

## Methods

### Patient population and treatment

Data from four RCTs (FER‐CARS‐01,^18^ FAIR‐HF,^16^ EFFICACY‐HF,^19^ and CONFIRM‐HF^17^) examining FCM's safety and efficacy in treatment of patients with CHF and ID were pooled on a patient level. All studies have been completed up to December 2014. For the model population, baseline information from 833 eligible patients with systolic HF and ID and baseline New York Heart Association (NYHA) functional class II/III participating in the trials were included in our analysis. The budget impact model (BIM) compares treatment of FCM with no‐iron, based on the pooled RCT data.

### Analytical framework

**Figure 1:**
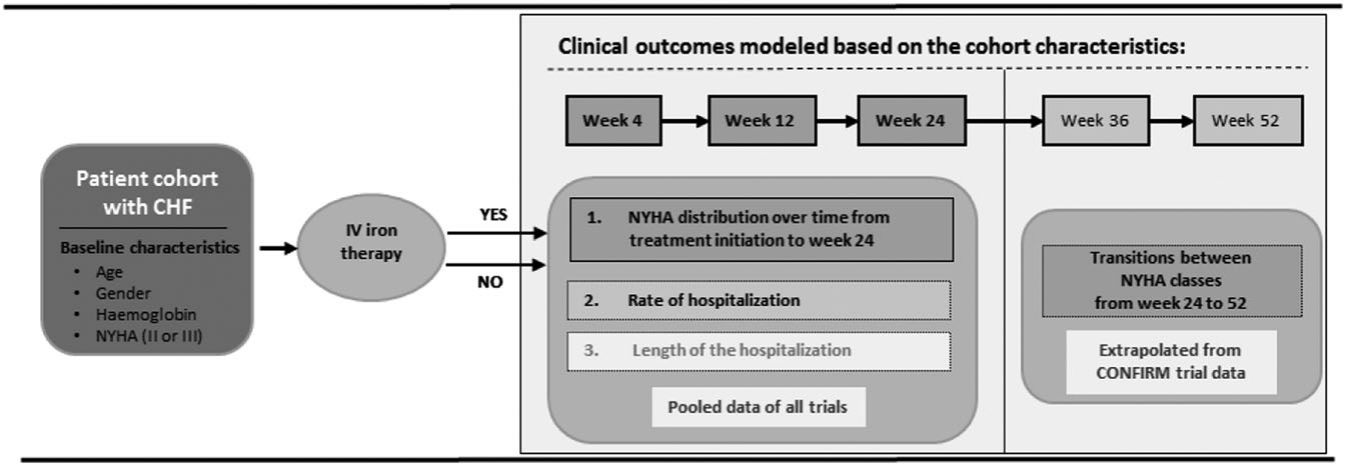
Model structure. CHF, chronic heart failure; i.v., intravenous; NYHA, New York Heart Association.

To estimate the budget impact of FCM in patients with CHF and ID compared with no‐iron, we developed a decision analytic BIM. Trial data were used to develop statistical models predicting the NYHA class distribution over time, rates of hospitalizations, and average length of stay (LOS) for all NYHA class I–IV. *Figure* *1* shows the model structure.

The modelled sample size for the base case is 1000 patients with CHF and ID per treatment arm, based on an extrapolation from the pooled trial data. For the base case, the chosen time horizon is 1 year. Because of different follow‐up times in the included trials, the pooled dataset was limited to 24 weeks of follow‐up. In order to extend the time horizon to 52 weeks, CONFIRM‐trial data alone are used to model the clinical outcomes to predict the population‐averaged transition probabilities from week 24 to week 36 and from week 36 to week 52 using repeated measures logistic regression models (generalized estimating equations, with logit‐link function, binomial distribution, and exchangeable correlation structure). The chosen perspective for the base case of the BIM is the SHI. Improvements in NYHA class and decrease in related health resource use as well as avoided hospitalizations due to HF worsening define the budget impact.

### Model inputs and statistics

#### Clinical parameter

The key clinical outcomes to inform the BIM are as follows: (i) probabilities to be in specific NYHA class (I–IV) or death over time; (ii) rate of hospitalization due to worsening of CHF; and (iii) average length of hospital stay (LOS). To predict these outcomes, we analysed the pooled data set with suitable multivariate statistical methods to find the best statistical fit. All prediction models implemented into the BIM included baseline age, gender, haemoglobin, and NYHA class. Geographical region was considered in the analyses and was included as predictor if the region variable improved model fit or model predictions. The variable selection for the statistical models were guided by information criteria (Akaike and Bayesian), log‐likelihood ratio test, and most importantly by the accuracy and credibility of the statistical model predictions. A significance level of *P* < 0.05 was considered significant. For all statistical analyses, STATA MP 64‐bit version 14.0 or later (StataCorp, College Station, TX, USA) commercial statistical software was used.

##### NYHA class distributions over time and number of deaths

To predict the disease status in terms of NYHA class over time, multinomial logistic regression model was built and implemented to the BIM. According to the RCTs, we assumed that, if the subject was hospitalized or had died at the time of the planned NYHA assessment, the subject was considered as having NYHA class IV or death, respectively. If an observation was missing and the subject were neither dead nor hospitalized at the time of the study visit on week 12 or 24 visits, the missing observations were imputed by carrying forward the last observation. The clustered nature of the data (repeated measures) was accounted for in the estimation. We established three separate statistical models predicting (i) improvement of NYHA class; (ii) worsening of NYHA class; and (iii) death after week 24 with the same covariates that were used in the final statistical model predicting NYHA class distribution over time up to 24 weeks.

##### Rate of hospitalization and average length of stay

Hospitalization rate due to worsening of CHF was estimated using a log‐link negative binomial regression model with length of observation included as an offset variable to adjust for the differing observation periods (to control for the length time bias) between patients and trials. The average LOS per hospitalization due to worsening of CHF was estimated using a log‐link negative binomial regression model. In order to obtain robust estimates, the multivariate analyses used the data from all cardiovascular hospitalizations with a variable for reason of hospitalization (other cardiovascular vs. worsening HF) included as an additional covariate.

#### Economic parameter

For the BIM, only direct costs were of interest. The costs include the disease related costs of hospitalization, medications, outpatient visits, home visits, and other healthcare resources. In the base case, we used the cost data from the German AnyCare Database. This database includes anonymized data from SHI services for CHF patients. The analysis sample covers reimbursed services and costs of 369 361 insured individuals located across Germany and is representative for the SHI population in gender and age.^20^, ^21^ Cost from the claims data base were inflation‐adjusted to 2014. The model estimates the costs of FCM for the mean cumulative dose of 1679 mg based on pooled data set multiplied by the calculated mean cost per milligram of FCM (€0.317). Price for FCM came from the official German drug price list (‘Lauertaxe’).^22^ For more information, see *Table* 1.

**Table 1:** Resource use and unit costs

Economic parameter	Value (annual unit cost in €)				Source
NYHA class	I	II	III	IV	
Price of FCM (1000 mg)	316.71^a^				CompuGroup Medical^22^
Cost of other medication per year	1201.53	1574.95	1921.53	2013.70	Federal Health Monitoring System^20^ and Anycare^21^, ^b^
Cost of outpatient visits per year	190.84	294.32	459.16	638.56	
Cost of home visits per year	11.44	20.28	49.40	141.44	
Cost of one hospitalization period	4141.00	4288.00	4744.00	6104.00	

FCM, ferric carboxymaltose; NYHA, New York Heart Association.

a List ex‐factory price excluding mandatory rebates.

b Costs were inflation‐adjusted to 2014.

### Sensitivity analysis

**Figure 2:**
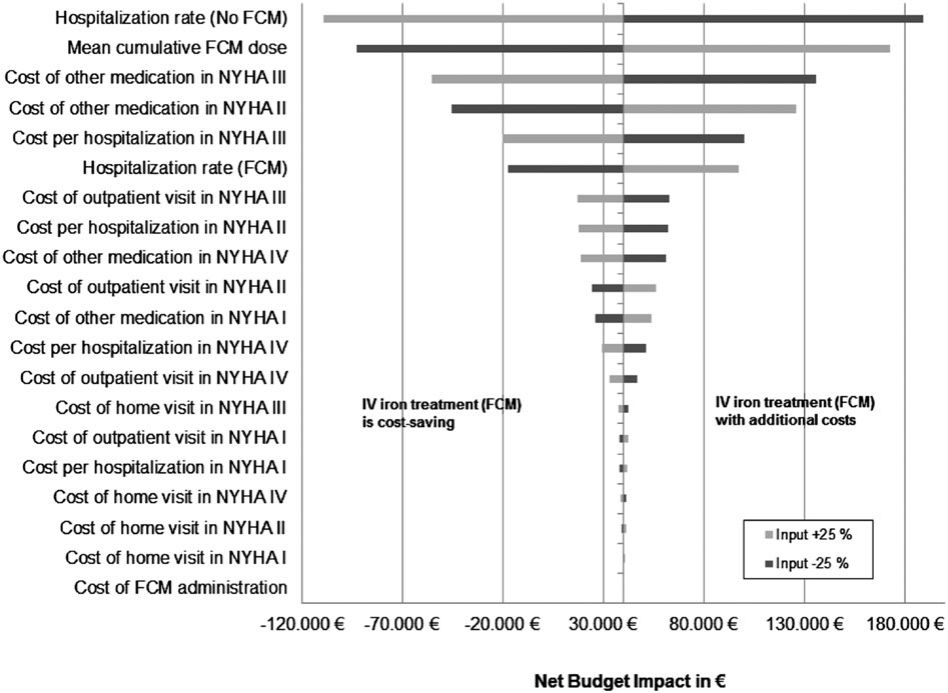
Results of the deterministic sensitivity analysis. FCM, ferric carboxymaltose; i.v., intravenous; NYHA, New York Heart Association.

Deterministic sensitivity analyses (DSA) were carried out to test the robustness of the results by varying the results ±25% as well as using reasonable published costs and resource uses from fee catalogues and other studies. Multiple extreme scenarios were run (*Table* A1). The results of the DSAs are also presented as a form of a tornado diagram, (*Figure* *2*) which depicts graphically how selected variations (default ±25%) in selected input affect the budget impact result. Each line represents the calculated impact if costs of the respective economic parameter are changed by 25% increase (white bars) or 25% decrease (black bar) of the assumed costs.

## Results

### Results of the pooled analysis

#### Population and treatment

Of the pooled trial participants, 33% and 67% were in the NYHA class II and III, respectively. Mean age was 68.1 years with balanced gender distribution (49% were women). Mean haemoglobin was 12.1 g/dL. The mean cumulative annual dose coming from the trials was 1679 mg for FCM treatment.

#### Predicted NYHA class distributions over time and number of deaths

According to the base case model, 33% of the patient cohort is in NYHA class II and 67% in NYHA class III before treatment. Following treatment (*Table* 2), more patients improved in NYHA class in the FCM‐treated group compared with no treatment. At week 52, 63.1% of the patients treated with FCM were in NYHA II and 23.5% in NYHA III. By contrast, in the ‘no‐iron’ group, more patients remained in NYHA class III (44.2%) than in NYHA II (39.2%). At week 52, 2.1% of the patients treated with FCM were predicted to be in NYHA IV, compared with 6.9% in patients in no‐iron group. The respective numbers for death at week 52 were 5.2% and 8.9% of the patients in FCM group and no‐iron treatment group, respectively.

**Table 2:** Predicted New York Heart Association distributions over time in the base case patient cohort

NYHA class	Treatment										Regression model^a^
	No‐iron (%)					FCM (%)					
	I	II	III	IV	Death	I	II	III	IV	Death	
Baseline	0	33	67	0	0	0	33	67	0	0	Multinomial logistic regression^b^
Week 4	0.1	30.1	65.6	4.0	0.2	0.9	51.6	46.0	1.4	0.1	
Week 12	0.4	35.9	57.2	4.2	2.2	5.1	55.9	36.4	1.3	1.3	
Week 24	0.5	43.3	45.8	6.3	4.1	5.4	63.1	27.3	1.9	2.3	
Week 36	0.7	41.1	45.1	6.6	6.5	5.8	63.2	25.2	2.0	3.8	Repeated measures logistic regression^c^
Week 52	0.9	39.2	44.2	6.9	8.9	6.1	63.1	23.5	2.1	5.2	

FCM, ferric carboxymaltose; NYHA, New York Heart Association.

a Regression models utilize baseline characteristics: age, gender, Haemoglobin, and baseline NYHA (III vs. II) were included in the models.

b Within patient clustering accounted in the variance estimator. Utilizes pooled data from all four randomized controlled trials.

c Generalized estimating equations model with logit‐link function, binomial distribution, and exchangeable correlation structure. Data for weeks 36 and 52 from CONFIRM‐trial.

#### Predicted rate of hospitalization and average length of stay

For the modelled 52 week time horizon, the FCM group had 52 hospitalizations per 1000 patients vs. 129 hospitalizations in the no‐iron group (*Table* 3). When considering the predicted average LOS in each treatment group, this corresponds to a total of 718 days and 2240 days of inpatient care over the 52 week period in FCM and no‐iron therapy groups, respectively.

**Table 3:** Predicted hospitalization rates and length of stay in the base case patient cohort

Clinical outcome	No‐iron	FCM	Regression model
Hospitalization rate^a^	0.0026	0.0010	Negative binomial regression
Average LOS^b^	17.40	13.85	Negative binomial regression

FCM, ferric carboxymaltose; LOS, length of stay.

a Number of hospitalizations per patient‐week. Predicted using baseline characteristics: age, gender, haemoglobin, New York Heart Association (III vs. II), and region. Only hospitalizations due to heart failure worsening included.

b Days per hospitalization period. Predicted using baseline age, gender, haemoglobin, New York Heart Association (III vs. II), and reason for hospitalizations (other cardiovascular vs. worsening of chronic heart failure).

### Results of the budget impact model

#### Economic outcome

Annual total costs of healthcare resources in our model for state‐of‐the‐art HF therapy without iron supplementation amounted to €2 694 474 per year for 1000 patients (*Table* 4).

**Table 4:** Modelled annual costs (€) of FCM and no‐iron per 1000 German CHF patients

Treatment cost driver	No‐iron	FCM	Net budget impact
Healthcare resources			
Home visits	40 469	29 627	−10 842
Outpatient visits	378 019	332 523	−45 496
Hospitalizations due to HF worsening	597 078	230 591	−366 487
CHF related medications	1 679 908	1 611 007	−68 900
Total cost of healthcare resources	2 695 474	2 203 749	−491 725
Total cost of FCM treatment	0	531 756	531 756
Total cost	2 695 474	2 735 505	40 031

CHF, chronic heart failure; FCM, ferric carboxymaltose; HF, heart failure.

The improved disease state (NYHA class) and fewer hospitalizations due to FCM therapy, however, resulted in reduced healthcare costs of €2 203 749 or savings of €491 724. The cumulated savings resulted from lower costs for hospitalization due to HF worsening (−€366 487), from reduced costs for other HF‐related medication (−€68 900), for outpatient visits (−€45 496), and for home visits (−€10 841). These cost savings on HF therapy were counterbalanced by costs for FCM treatment totalling at €531 756.

The base case according to the model has an annual budget impact of €40 031 per 1000 patients for intravenous (i.v.) iron substitution using FCM compared with no‐iron during the first year after initiation of FCM treatment. In other words, i.v. iron therapy accounts for additional costs of €40 per patient per year. These costs are the financial equivalent of 1522 patient‐days saved from hospitalization per 1000 patients and 36.2% of patients being improved by at least one NYHA class in the FCM group compared with 7.1% in the no‐iron group.

#### Sensitivity analysis

The DSA Tornado diagram in *Figure* *2* shows the most important financial factors influencing the base case analysis, starting from the base case (i.e. €40 032 per 1000 patients).

The change of the hospitalization rate in the no‐iron group has the highest impact on the healthcare budget. Assuming higher hospitalization costs by 25% would mean additional costs savings by FCM therapy of −€109 238, and 25% lower hospitalization costs mean increased expenses for FCM therapy of €189 301. Following cost factors are change of mean cumulative FCM dose (+25%: €172 971; −25%: −€92 907) and the change of costs of other medication in NYHA class III (+25%: −€55 745; −25%: €135 809). When only treating patients in NYHA class III or II, therapy with FCM leads to net budget impact of −€110 380 or €321 137 compared with no‐iron treatment. Assuming the same clinical effects and using real‐world data for mean FCM dose of 925 mg, taken from a real‐world study by Bierbaum *et al.*,^23^ the annual FCM costs will be €292 957, and the net budget impact changes to −€198 768 demonstrating cost savings. Changing the FCM price by 25%, the budget impact ranges from €173 495 (price +25%) to −€92 626 (price −25%). Detailed results are shown in Appendix 1.

## Discussion

This is the first study to analyse the budget impact of treatment of ID using FCM in patients with HF in the German public healthcare setting. Our analysis model shows that the treatment with FCM is associated with minimal budget impact over the first year of FCM treatment. On average, patients treated with FCM were predicted to have better symptomatic disease status and fewer and shorter hospitalizations for HF compared with patients without iron therapy. Clinical benefit was observed already within 4 weeks of therapy^16^ and persisted throughout the 52 week treatment period.^17^ Consistent with Ponikowski *et al.*,^24^ the rate of hospitalized patients was significantly lower in the FCM‐treated patient group. The cost category with the highest contribution to the estimated costs was ‘CHF‐related medications’ (FCM group €1 611 007 vs. no‐iron group €1 679 908, *Table* 4). Nevertheless, the highest potential for savings was for the FCM‐treated group in the category ‘Hospitalizations due to HF worsening’ (−€366 487). Compared with the patients with no‐iron therapy, the FCM treated group had additional expenditures for the i.v. iron supplementation with FCM (€531 756). Summarizing all net costs in the base case model, the incremental treatment cost for FCM compared with no‐iron treatment were only 40 per patient during the first year of treatment.

Walter *et al.* 25 developed a cost‐utility analysis utilizing a 4 year time horizon for an Austrian setting, based on the pivotal FCM studies FAIR‐HF and CONFIRM‐HF. They found that FCM treatment is clearly below the National Institute for Health and Care Excellence (NICE) cost‐effectiveness threshold of €22 000–33 000/quality adjusted life year when comparing FCM with oral iron treatment in CHF patients with ID and without anaemia. Gutzwiller *et al.* 26 also modelled cost‐effectiveness from the perspective of a UK payer using the FAIR‐HF data. Their analysis only modelled the time horizon of 24 weeks but showed similar conclusions. Comin‐Colet *et al.* 27 came to the same results in their cost‐effectiveness analysis in Spain. They used the data of the FAIR‐HF trial and took only the time horizon of 24 weeks. A Swedish analysis based on FAIR‐HF trial concluded that treatment of ID in CHF with FCM compared with placebo is estimated to be cost‐effective. The base case scenario is noticeably below SEK 500 000 (€54 300) per quality adjusted life year, an informal average reference value used by the Swedish Dental and Pharmaceutical Benefits Agency.^28^ In comparison to these analysis, in our study, we utilized the available data of 52 week therapy in the CONFIRM‐HF study, which allowed for the longer time horizon of 52 weeks. We also used more comprehensive efficacy data in our model by employing patient‐level data of four FCM trials.

For the Swiss Health Care System, Brock *et al.* 29 evaluated all ID‐related diagnoses including CHF to show the impact of FCM treatment over a 6 year period. They used unpublished data from different databases (e.g. IMS, prescriber analysis, ‘Anaemia Patient Record Study’) but not from clinical trials. In all clinical indications except in patients with dialysis, treatment with FCM led to cost savings. Overall, it seems problematic to compare the results from these individual health economic studies. Different patient populations were studies, and different types of modelling were applied. Nevertheless, our study is in agreement with the existing economic evaluations in other countries all confirming advantages for FCM treatment.^15^

### Limitations

One of the key limitations of the present budget impact analysis is that all studies included in the data analyses were placebo‐controlled, double blind, multinational, multicentre RCTs and may not reflect the real‐world treatment patterns and effectiveness in the German healthcare setting. Wherever they had impact on the model fit or predictions, the regional differences were accounted for in the multivariate prediction models. Unfortunately, specific subgroup analysis for the German population could not be performed due to the small sample size.

Mean cumulative dose used in the model was 1679 mg given in 2.1 applications. Assuming annual real‐life dose of 925 mg per patient, taken from a real‐world study of Bierbaum *et al.*,^23^ the annual FCM costs would decrease by 45% per patient and treatment with FCM would be net cost saving compared with no‐iron. Excluding the usual €10 co‐payment by the patients himself for a 1000 mg package of FCM, the price would decrease to €306.71, and budget impact of FCM treatment would decrease to €23 per treated patient compared with a no‐iron therapy. Besides this, the baseline distribution of NYHA class and effectiveness might be different in a real‐world setting.

A recently published cost comparison for Germany using accounting data from the pharmacy data processing centres^23^ concluded that treatment with FCM is less costly than using other i.v. iron compounds. Also, extreme scenario analysis shows that, when only treating NYHA class III or II CHF per 1000 patients, therapy with FCM leads to cost savings (i.e. −€110 380) or additional costs (i.e. €321 137) compared with no‐iron treatment, respectively.

Our approach for the base case (perspective of the SHI) does not account for the predicted LOS reduction or the improved productivity, which is associated with reduced number of deaths and improved functional capacity of patients. In turn, we did not account for the costs per dead individual because we do not know exactly the cost of death in Germany. Based on the trial data, treatment with FCM is associated with substantial reduction in average LOS [on average (2240/129) − (718/52) = 3.6 days per hospitalization]. From the health economic perspective as from the hospital manager perspective, treatment with FCM resulted in cost savings.

The healthcare costs obtained from the claims data analysis are inflation‐adjusted because up‐to‐date cost data were not available for CHF+ID patients. These costs are reimbursed fees from a SHI data sample with limited observations (*N*_with NYHA I–IV_ = 4989) and without considering any co‐payments. It may be argued that this sample may not reflect the whole SHI population. However, because of our conservative approach, all cost‐relevant services included in the model only differ by NYHA class and not between the treatment arms. This might be different in a real‐world setting.

Our analysis was restricted to direct healthcare‐related costs and at a 1 year time horizon, not extrapolating the efficacy predictions or cost impacts beyond the available trial data. Other economic evaluations may cover a longer time period and take a wider perspective or the social perspective (e.g. including productivity costs).

## Conclusions

In conclusion, ID is a relevant comorbidity in HF associated with a worse symptomatic status and increased hospitalization. Treating this complication by i.v. supplementation of iron results in improved symptomatic status and reduced frequency and duration of hospitalization. This clinical benefit is achieved by additional costs of a mere 40 € per patient per year. Our study confirms that treatment with FCM addresses the unmet medical need in patients with HF and ID patients at minimum economic impact to the German public healthcare system.

## Conflict of Interest

U.T. received fees from Vifor Pharma GmbH and other companies because of consultancy work for Xcenda GmbH.

S.V. is an employee of ESiOR Oy, a consultancy company serving Xcenda and other companies.

J.M. is an employee and holds stock of ESiOR Oy, a consultancy company serving Xcenda and other companies.

E.S. is an employee and holds stock of ESiOR Oy, a consultancy company serving Xcenda and other companies.

T.H. is an employee of Vifor Pharma Germany.

W.D. received honoraria and research funding from Vifor Pharma.

## Funding

Vifor Pharma GmbH, Munich, Germany.

## Appendix

**Table A1:** Overview for diverse extreme scenario analysis (all costs in € per 1000 patients)

Parameters varied	No‐iron	FCM	Net budget impact (€)
Base case	2 695 474	2 735 505	40 032
Proportion of NYHA II = 100%	2 191 435	2 512 572	321 137
Proportion of NYHA III = 100%	3 116 291	3 005 911	−110 380
Average haemoglobin lower limit (9.9 g/dL)	2 759 981	2 751 578	−8 403
Average haemoglobin upper limit (14.3 g/dL)	2 608 996	2 701 725	92 729
Proportion of men = 100%	2 853 825	2 769 679	−84 146
Proportion of women = 100%	2 582 260	2 719 841	137 580
Age lower limit (50 years on average)	2 514 056	2 652 635	138 579
Age upper limit (82 years on average)	2 724 631	2 734 772	10 142
Real‐world FCM dose by Bierbaum *et al.* 23	2 695 474	2 496 706	−198 768
Costs by Biermann *et al.* 10 (adjusted to 2014)	859 362	1 211 713	352 351
Costs by Zugck *et al.* 11 (adjusted to 2014)	3 600 314	3 286 292	−314 022
Lump sums from fee catalogues^a^	654 138	945 372	353 080
Account for the predicted LOS reduction	3 030 138	2 803 548	−226 590

FCM, ferric carboxymaltose; LOS, length of stay; NYHA, New York Heart Association.

a EBM 03000/03220 (€29.45) for general practitioner visits and DRG F62A/B (weighted average €2969.04) for hospitalization.

## References

[1] Kemp CD , Conte JV . The pathophysiology of heart failure. Cardiovascular Pathology : the Official Journal of the Society for Cardiovascular Pathology 2012; 21: 365–371. 22227365 10.1016/j.carpath.2011.11.007

[2] Ponikowski P , Anker SD , AlHabib KF , Cowie MR , Force TL , Hu S , Jaarsma T , Krum H , Rastogi V , Rohde LE , Samal UC , Shimokawa H , Budi Siswanto B , Sliwa K , Filippatos G . Heart failure: preventing disease and death worldwide. ESC Heart Failure 2014; 1: 4–25. 28834669 10.1002/ehf2.12005

[3] Nieminen MS , Dickstein K , Fonseca C , Serrano JM , Parissis J , Fedele F , Wikstrom G , Agostoni P , Atar S , Baholli L , Brito D , Colet JC , Edes I , Gomez Mesa JE , Gorjup V , Garza EH , Gonzalez Juanatey JR , Karanovic N , Karavidas A , Katsytadze I , Kivikko M , Matskeplishvili S , Merkely B , Morandi F , Novoa A , Oliva F , Ostadal P , Pereira‐Barretto A , Pollesello P , Rudiger A , Schwinger RH , Wieser M , Yavelov I , Zymlinski R . The patient perspective: quality of life in advanced heart failure with frequent hospitalisations. Int J Cardiol 2015; 191: 256–264. 25981363 10.1016/j.ijcard.2015.04.235

[4] Peters‐Klimm F , Kunz CU , Laux G , Szecsenyi J , Muller‐Tasch T . Patient‐ and provider‐related determinants of generic and specific health‐related quality of life of patients with chronic systolic heart failure in primary care: a cross‐sectional study. Health Qual Life Outcomes 2010; 8: 98. 20831837 10.1186/1477-7525-8-98 PMC2945966

[5] Pisa G , Eichmann F , Hupfer S . Assessing patient preferences in heart failure using conjoint methodology. Patient Prefer Adherence 2015; 9: 1233–1241. 26345530 10.2147/PPA.S88167 PMC4556263

[6] Ohlmeier C , Mikolajczyk R , Frick J , Prutz F , Haverkamp W , Garbe E . Incidence, prevalence and 1‐year all‐cause mortality of heart failure in Germany: a study based on electronic healthcare data of more than six million persons. Clin Res Cardiol 2015 Aug; 104: 688–696. 25777937 10.1007/s00392-015-0841-4

[7] Statistisches Bundesamt . Die 20 häufigsten diagnosen. https://www.destatis.de/DE/ZahlenFakten/GesellschaftStaat/Gesundheit/Krankenhaeuser/Tabellen/Diagnosen.html;jsessionid=1878304D3BB52F659B91BC1CC5314860.cae4 (17 February 2016).

[8] Statistisches Bundesamt . Todesursachenstatistik. https://www.destatis.de/DE/Publikationen/Thematisch/Gesundheit/Todesursachen/Todesursachenstatistik.html (17 February 2016).

[9] Neumann T , Biermann J , Erbel R , Neumann A , Wasem J , Ertl G , Dietz R . Heart failure: the commonest reason for hospital admission in Germany: medical and economic perspectives. Dtsch Arztebl Int 2009; 106: 269–275. 19547628 10.3238/arztebl.2009.0269 PMC2689573

[10] Biermann J , Neumann T , Angermann CE , Erbel R , Maisch B , Pittrow D , Regitz‐Zagrosek V , Scheffold T , Wachter R , Gelbrich G , Wasem J , Neumann A , German Competence Network Heart F . Economic burden of patients with various etiologies of chronic systolic heart failure analyzed by resource use and costs. Int J Cardiol 2012; 156: 323–325. 22373485 10.1016/j.ijcard.2012.01.099

[11] Zugck C , Muller A , Helms TM , Wildau HJ , Becks T , Hacker J , Haag S , Goldhagen K , Schwab JO . Health economic impact of heart failure: an analysis of the nationwide German database. Dtsch Med Wochenschr 2010 Apr; 135: 633–638. 20333603 10.1055/s-0030-1251912

[12] Klip IT , Comin‐Colet J , Voors AA , Ponikowski P , Enjuanes C , Banasiak W , Lok DJ , Rosentryt P , Torrens A , Polonski L , van Veldhuisen DJ , van der Meer P , Jankowska EA . Iron deficiency in chronic heart failure: an international pooled analysis. Am Heart J 2013 Apr; 165: 575–582 e573. 23537975 10.1016/j.ahj.2013.01.017

[13] Krum H , Gilbert RE . Demographics and concomitant disorders in heart failure. Lancet 2003 Jul 12; 362(9378): 147–158. 12867118 10.1016/S0140-6736(03)13869-X

[14] McMurray JJ , Adamopoulos S , Anker SD , Auricchio A , Bohm M , Dickstein K , Falk V , Filippatos G , Fonseca C , Gomez‐Sanchez MA , Jaarsma T , Kober L , Lip GY , Maggioni AP , Parkhomenko A , Pieske BM , Popescu BA , Ronnevik PK , Rutten FH , Schwitter J , Seferovic P , Stepinska J , Trindade PT , Voors AA , Zannad F , Zeiher A , Guidelines ESCCfP . ESC Guidelines for the diagnosis and treatment of acute and chronic heart failure 2012: the Task Force for the Diagnosis and Treatment of Acute and Chronic Heart Failure 2012 of the European Society of Cardiology. Developed in collaboration with the Heart Failure Association (HFA) of the ESC. Eur Heart J 2012; 33: 1787–1847. 22611136 10.1093/eurheartj/ehs104

[15] Ponikowski P , Voors AA , Anker SD , Bueno H , Cleland JG , Coats AJ , Falk V , Gonzalez‐Juanatey JR , Harjola VP , Jankowska EA , Jessup M , Linde C , Nihoyannopoulos P , Parissis JT , Pieske B , Riley JP , Rosano GM , Ruilope LM , Ruschitzka F , Rutten FH , van der Meer P , Authors/Task Force M, Document R. 2016 ESC Guidelines for the diagnosis and treatment of acute and chronic heart failure: the Task Force for the Diagnosis and Treatment of Acute and Chronic Heart Failure of the European Society of Cardiology (ESC). Developed with the special contribution of the Heart Failure Association (HFA) of the ESC. Eur Heart J 2016 May; 20: 39–40. 10.1002/ejhf.592 27207191

[16] Anker SD , Comin Colet J , Filippatos G , Willenheimer R , Dickstein K , Drexler H , Luscher TF , Bart B , Banasiak W , Niegowska J , Kirwan BA , Mori C , von Eisenhart RB , Pocock SJ , Poole‐Wilson PA , Ponikowski P , Investigators F‐HT . Ferric carboxymaltose in patients with heart failure and iron deficiency. N Engl J Med 2009 Dec 17; 361: 2436–2448. 19920054 10.1056/NEJMoa0908355

[17] Ponikowski P , van Veldhuisen DJ , Comin‐Colet J , Ertl G , Komajda M , Mareev V , McDonagh T , Parkhomenko A , Tavazzi L , Levesque V , Mori C , Roubert B , Filippatos G , Ruschitzka F , Anker SD , Investigators C‐H . Beneficial effects of long‐term intravenous iron therapy with ferric carboxymaltose in patients with symptomatic heart failure and iron deficiencydagger. Eur Heart J 2015 Mar 14; 36: 657–668. 25176939 10.1093/eurheartj/ehu385 PMC4359359

[18] Arutyunov G . The safety of intravenous (IV) ferric carboxymaltose versus IV iron sucrose on patients with chronic heart failure (CHF) and chronic kidney disease (CKD) with iron deficincy (ID). Eur J Heart Fail 2009; 8: ii71.

[19] Effect of ferric carboxymaltose on exercise capacity and cardiac function in patients with iron deficiency and chronic heart failure (EFFICACY‐HF) (data on file). U.S. National Institutes of Health. https://clinicaltrials.gov/ct2/show/NCT00821717 (5 April 2017).

[20] Mitglieder und mitversicherte familienangehörige der gesetzlichen krankenversicherung am 1.7. eines jahres (Anzahl). Gliederungsmerkmale: Jahre, Deutschland, Alter, Geschlecht, Kassenart, Versichertengruppe. Gesundheitsberichterstattung des Bundes. www.gbe‐bund.de (13 April 2016).

[21] Anycare . Versorgungsforschung anycare: herzinsuffizienz (Auswertungsjahr 2011). Data on file 2013.

[22] Preisinformation ferinject 50mg eisen/ml. Lauertaxe. http://www2.lauer‐fischer.de/home/ (16 November 2015).

[23] Bierbaum M , Schöffski O . Kosten‐Kosten‐Studie zur parenteralen eisentherapie bei eisenmangelanämie in der ambulanten versorgung der GKV in Deutschland. Gesundh ökon Qual manag 2013; 18: 173–179.

[24] Rocca HP , Crijns HJ . Iron i.v. in heart failure: ready for implementation? Eur Heart J 2015; 36: 645–647. 25336211 10.1093/eurheartj/ehu392 PMC4359360

[25] Walter E , Bauer M , Ressl S . Cost‐effectiveness of ferric carboxymaltose in patients with Iron deficiency and chronic heart failure in Austria. Value Health 2015; 18: A392.

[26] Gutzwiller FS , Schwenkglenks M , Blank PR , Braunhofer PG , Mori C , Szucs TD , Ponikowski P , Anker SD . Health economic assessment of ferric carboxymaltose in patients with iron deficiency and chronic heart failure based on the FAIR‐HF trial: an analysis for the UK. Eur J Heart Fail 2012; 14: 782–790. 22689292 10.1093/eurjhf/hfs083 PMC3380546

[27] Comin‐Colet J , Rubio‐Rodriguez D , Rubio‐Terres C , Enjuanes‐Grau C , Gutzwiller FS , Anker SD , Ponikowski P . A cost‐effectiveness analysis of ferric carboxymaltose in patients with iron deficiency and chronic heart failure in Spain. Rev Esp Cardiol (Engl Ed) 2015; 68: 846–851. 25649970 10.1016/j.rec.2014.10.010

[28] Hofmarcher T , Borg S . Cost‐effectiveness analysis of ferric carboxymaltose in iron‐deficient patients with chronic heart failure in Sweden. J Med Econ 2015; 18: 492–501. 25766863 10.3111/13696998.2015.1029491

[29] Brock E , Braunhofer P , Troxler J , Schneider H . Budget impact of parenteral iron treatment of iron deficiency: methodological issues raised by using real‐life data. Eur J Health Econ 2014; 15: 907–916. 24081613 10.1007/s10198-013-0533-9

